# Determinants of early-life lung function in African infants

**DOI:** 10.1136/thoraxjnl-2015-207401

**Published:** 2016-11-17

**Authors:** Diane Gray, Lauren Willemse, Ane Visagie, Dorottya Czövek, Polite Nduru, Aneesa Vanker, Dan J Stein, Nastassja Koen, Peter D Sly, Zoltán Hantos, Graham L Hall, Heather J Zar

**Affiliations:** 1 Department of Paediatrics and Child Health, Red Cross War Memorial Children's Hospital and MRC Unit on Child and Adolescent Health, University of Cape Town, Cape Town, South Africa; 2 Children's Lung, Environment and Asthma Research, Child Health Research Centre, University of Queensland, Brisbane, Queensland, Australia; 3 Division of Epidemiology and Biostatistics, Department of Public Health and Family Medicine, University of Cape Town, Cape Town, South Africa; 4 Department of Psychiatry and MRC Unit on Anxiety and Stress Disorder, University of Cape Town, Cape Town, South Africa; 5 Department of Medical Physics and Informatics, University of Szeged, Szeged, Hungary; 6 Department of Pulmonology, University of Szeged, Szeged, Hungary; 7 Telethon Kids Institute, Australia Centre for Child Health Research, University of Western Australia, Perth, Western Australia, Australia; 8 Faculty of Health Sciences, School of Physiotherapy and Exercise Science, Curtin University, Perth, Western Australia, Australia

**Keywords:** Paediatric Lung Disaese, Respiratory Measurement, Tobacco and the lung, Clinical Epidemiology

## Abstract

**Background:**

Low lung function in early life is associated with later respiratory illness. There is limited data on lung function in African infants despite a high prevalence of respiratory disease.

**Aim:**

To assess the determinants of early lung function in African infants.

**Method:**

Infants enrolled in a South African birth cohort, the Drakenstein child health study, had lung function measured at 6–10 weeks of age. Measurements, made with the infant breathing via a facemask during natural sleep, included tidal breathing, sulfur hexafluoride multiple breath washout and the forced oscillation technique. Information on antenatal and early postnatal exposures was collected using questionnaires and urine cotinine. Household benzene exposure was measured antenatally.

**Results:**

Successful tests were obtained in 645/675 (95%) infants, median (IQR) age of 51 (46–58) days. Infant size, age and male gender were associated with larger tidal volume. Infants whose mothers smoked had lower tidal volumes (−1.6 mL (95% CI −3.0 to −0.1), p=0.04) and higher lung clearance index (0.1 turnovers (95% CI 0.01 to 0.3), p=0.03) compared with infants unexposed to tobacco smoke. Infants exposed to alcohol in utero or household benzene had lower time to peak tidal expiratory flow over total expiratory time ratios, 10% (95% CI −15.4% to −3.7%), p=0.002) and 3.0% (95% CI −5.2% to −0.7%, p=0.01) lower respectively compared with unexposed infants. HIV-exposed infants had higher tidal volumes (1.7 mL (95% CI 0.06 to 3.3) p=0.04) compared with infants whose mothers were HIV negative.

**Conclusion:**

We identified several factors including infant size, sex, maternal smoking, maternal alcohol, maternal HIV and household benzene associated with altered early lung function, many of which are factors amenable to public health interventions. Long-term study of lung function and respiratory disease in these children is a priority to develop strategies to strengthen child health.

**Key messages**

**What is the key question?**

- What are the antenatal and early-life determinants of infant lung function?

**What is the bottom line?**

- In addition to known factors such as growth, sex and maternal smoking, maternal alcohol during pregnancy, exposure to household benzene and having an HIV-positive mother alter lung function in early life.

**Why read on?**

- Low lung function in early life is associated with later respiratory disease. Understanding what impacts early lung function is critical in strengthening our understanding of respiratory disease risk, especially in high burden settings.

## Background

Lower respiratory tract infections (LRTIs) are the leading cause of death in children in low/middle-income countries (LMICs),^1^ and chronic respiratory illness is a common sequela.^2^ There is increasing evidence that chronic respiratory disease in later life has its origins in childhood, with low lung function in early life being associated with lung function impairment and chronic respiratory illness in later life.^3^ Identifying prenatal and postnatal factors associated with early lung disease, as measured by lung function, will provide a better understanding of how early-life exposures influence childhood lung disease and possibly provide insights into prevention of subsequent chronic respiratory illness. The normal development of the human lung begins in utero but continues in postnatal life until adolescence. Thus, in utero and early-life factors that damage or impair lung growth may have a considerable impact on early lung function.

Several risk factors for altered early lung function or respiratory disease in childhood have been identified including infant birth weight,^4^ prematurity,^5^ early-life respiratory tract infections,^6^ maternal asthma^7^ and environmental factors such as tobacco smoke exposure^8^ and household air pollution.^9^ Many of these factors are common in Africa but there are no data on early lung function in these settings despite the high burden of childhood respiratory disease.

We aimed to investigate the impact of antenatal and early-life exposures on lung function measured at 6 weeks of age in African infants enrolled in a birth cohort.

## Method

### Setting

Infants enrolled in a birth cohort study, the Drakenstein Child Health study,^10^ had lung function tested. This study, set in a periurban, low socioeconomic community in South Africa, aims to investigate the epidemiology and aetiology of childhood respiratory illness and the determinants of child health. Participants were enrolled at two primary care clinics, Mbekweni, serving a predominantly black African population and Newman, serving a predominantly mixed ancestry population. Lung function testing was undertaken at the local hospital.

### Participants

Infants underwent testing at 5–11 weeks of age corrected for prematurity (37 weeks). Infants born <32 weeks gestation or with congenital anomalies were excluded from this analysis. Mothers had spirometric lung function (Jaeger Masterscope, CareFusion, Switzerland) at the same visit, provided they had not had a respiratory infection within the last 2 weeks.

### Exposures

Information regarding antenatal, birth and early-life exposures and events were collected by questionnaire at scheduled antenatal and study visits. These are comprehensively defined in online supplementary table S1. The socioeconomic status (SES) was defined in quartiles from lowest to highest status. This score was derived from employment status and standardised scores of educational attainment, household income, assets and market access (bank accounts, shops accessed, retail accounts); this methodology has been validated for capturing SES variation within an LMIC setting.^11^ LRTI was defined according to WHO criteria,^12^ and based on confirmatory examination by trained study staff (professional nurse and/or doctor).

10.1136/thoraxjnl-2015-207401.supp1 supplementary data

Maternal recurrent respiratory symptoms or low lung function was defined as at least one of doctor diagnosed asthma, chronic cough or recurrent wheeze in previous 12 months and/or low FEV_1_, defined as FEV_1_ < −1.64 SDs, predicted using the Global Lung Initiative multiethnic equations.^13^ Maternal distress and alcohol use during pregnancy were assessed using the self reporting questionnaire (SRQ) −20 and Alcohol, Smoking and Substance Involvement Screening Test (ASSIST) self-reported questionnaires completed at 28–32 weeks gestation.^14^ The SRQ-20 is a widely used WHO-endorsed measure of psychological distress.^15^ We used a dichotomous score of ‘high risk’ versus ‘low risk’, with ‘high risk’ defined as a score of ≥8.^15^ Infants were classified as alcohol-exposed in utero if their mother reported ASSIST scored heavy exposure with high risk for alcohol related problems: daily and/or weekly use of alcohol during at least 3 months of pregnancy.

Maternal smoking history was corroborated on a quantitative analysis of maternal urine cotinine (IMMULITE 1000 Nicotine Metabolite Kit; Siemens Medical Solutions Diagnostics, Glyn Rhonwy, UK) collected antenatally and at birth. Smoking exposure based on urine cotinine was defined as follows: active smoker if urine cotinine >500 ng/mL, passive smoker if urine cotinine 10–500 ng/mL and non-smoker if urine cotinine <10 ng/mL.^16^ Maternal urine was collected for cotinine testing at the second antenatal study visit (28–32 weeks gestation) and at birth, with the higher result used to classify smoking levels. Benzene, a household air pollutant, was measured at an antenatal home visit using a Markes thermal desorption tube left in the home for 2 weeks.^17^ The South African National Ambient Air Quality standard of 5 μg/m^3^ was used to define above and below threshold values for benzene.^17^

### Infant lung function measurements

Lung function measurements included tidal breathing and flow volume loops (TBFVL), sulfur hexafluoride (SF6) multiple breath washout (MBW) and the forced oscillation technique (FOT). Infants were tested from July 2012 to December 2014 for TBFVL and MBW and, for operational reasons, from October 2012 to December 2014 for FOT. Lung function was measured in unsedated infants during quiet sleep and conformed to American Thoracic society/European Thoracic society (ATS/ERS) guidelines,^18^ ^19^ as previously published.^20^ ^21^

Tidal breathing measures of tidal volume (V_T_), respiratory rate and expiratory flow ratios were collected using the Exhalyser D with ultrasonic flow metre (Ecomedics, Duernton, Switzerland) and analysed using analysis software (WBreath V.3.28.0; Ndd Medizintechnik, Zurich, Switzerland), as described previously.^20^ MBWs measuring the functional residual capacity (FRC) and lung clearance index (LCI) were performed using 4% SF6 as a tracer gas and ultrasonic flow metre (Spirison, Ecomedics) with acquisition and analysis software (WBreath V3.28.0, Ndd Medizintechnik) as reported previously.^22^ Measurements of respiratory system resistance (R_RS_) and compliance (C_RS_) with the FOT were made with purpose built equipment (University of Szeged, Hungary) using a medium frequency signal, as previously reported.^21^ ^23^

### Ethics

The study was approved by the Faculty of Health Sciences, Human Research Ethics Committee, University of Cape Town (401/2009) and by the Western Cape Provincial Health Research Committee. Mothers gave written informed consent in their first language for participation.

### Statistical analysis

Lung function outcomes were modelled using multiple linear regression to assess the impact of different antenatal and early-life exposures on lung function at 6–10 weeks. A base model was constructed using Directed Acyclic Graph (DAG) for confounder selection using graphical interface software DAGitty (http://www.dagitty.net V.2.2, 2014), (see online supplementary figure S2).^24^ DAG minimal adjusted set of variables were selected using a step-by-step approach.^25^

Interactions were then explored between infant growth and lung maturation (weight for age z score, gestation, birth weight z score), sex, ethnicity, environmental and socioeconomic factors (tobacco smoke exposure, high household benzene, SES), maternal factors (maternal stress score, infant feeding, maternal respiratory health, maternal HIV, antenatal alcohol) and previous LRTI, for each lung function outcome separately. Confounders and interactions were included in the final model for each outcome if they were associated with p value of <0.5 and/or the association had biological plausibility based on previous literature, as shown in online supplementary tables S2–S10.

Statistical analyses were performed using STATA V.13 for windows (STATA, College Station, Texas, USA). Data are presented as mean and SD for normally distributed variables and median and IQR for non-normally distributed variables. Weight (WAZ) and height (HAZ) for age z scores were calculated using the WHO Child Growth Standards ‘I grow up’ STATA package.^26^

## Results

**Figure 1:**
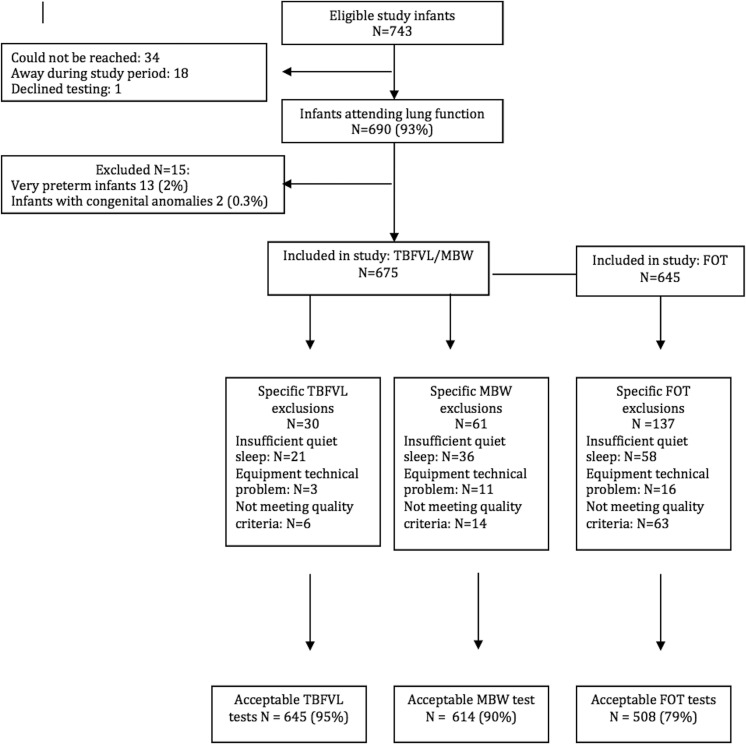
Description of cohort. FOT, forced oscillation technique; MBW, multiple breath washout; TBFVL, tidal breathing and flow volume loops.

Between July 2012 and December 2014, 690 infants were tested, of whom 15 were excluded as they were very preterm (13 (2%)) or had congenital abnormalities (2 (0.3%)) (figure 1). Infant testing was done at a median (IQR) of 51 (46–58) days, and anthropometry of enrolled infants is shown in table 1.

**Table 1:** Anthropometry by enrolment site

	Mbekweni (African black ethnicity), N=329	Newman (mixed ethnicity), N=346	Total (N=675)	p Value*
	Median (IQR)	Median (IQR)	Median (IQR)	
Age, days	50 (46 to 57)	52 (47 to 59)	51 (46 to 58)	0.1
Weight, kg	4.9 (4.4 to 5.5)	4.7 (4.2 to 5.3)	4.9 (4.3 to 5.4)	0.004
Weight for age z score	−0.2 (−0.8 to 0.6)	−0.6 (−1.4 to 0.1)	−0.4 (−1.2 to 0.4)	<0.001
Length, cm	56 (53 to 57)	55 (53 to 57)	55 (53 to 57)	0.08
Height for age z score	−0.7 (−1.7 to 0.3)	−1.0 (−2.0 to 0.0)	−0.8 (−1.8 to 0.1)	0.003
Weight for height z score	0.7 (−0.1 to 1.6)	0.5 (−0.3 to 1.3)	0.6 (−0.2 to 1.4)	0.010
Birth weight, kg	3.1 (2.8 to 3.4)	3.0 (2.6 to 3.4)	3.0 (2.7 to 3.4)	0.02
Z score birth weight	−0.4 (−1.3 to 0.3)	−0.8 (−1.5 to −0.1)	−0.6 (−1.4 to 0.1)	<0.001
Gestation weeks	39 (38 to 40)	39 (38 to 40)	39 (38 to 40)	0.7
Late preterm, 32–37 weeks, n (%)	46 (14.0)	40 (11.6)	86 (12.8)	0.345

*Kruskal-Wallis or Pearson χ^2^ tests comparing sites, Z scores calculated using the WHO Child Growth Standards^26^ and updated Fenton newborn growth charts.^27^

Infants from Mbekweni, who were predominantly black African infants, were of lower SES, less likely to have been breastfed and had higher HIV exposure compared with mixed ancestry infants, who had higher rates of tobacco smoke exposure (table 2).

**Table 2:** Demographics and socioeconomic factors of infants tested by enrolment site

	Mbekweni, N=329	Newman, N=346	Total, N=675	p Value
	n (%)	n (%)	n (%)	
Sex: male	157 (47.7)	197 (56.9)	354 (52.4)	0.032
African black	325 (98.8)	3 (0.9)	328 (48.6)	<0.001
Maternal smoking				
Non-smoker	100 (30.4)	39 (11.3)	139 (20.6)	<0.001
Passive exposure	167 (50.8)	131 (37.9)	298 (44.2)	
Smoker	50 (15.2)	168 (48.6)	218 (32.3)	
Unknown	12 (3.7)	8 (2.3)	20 (2.9)	
Household air pollution				
High benzene	141 (42.9)	129 (37.3)	270 (40.0)	0.262
Socioeconomic status				
Low	127 (38.6)	64 (18.5)	191 (28.3)	<0.001
Low moderate	84 (25.5)	82 (23.7)	166 (24.6)	
Moderate high	63 (19.2)	89 (25.7)	152 (22.5)	
High	55 (16.7)	110 (31.8)	165 (24.4)	
Breast feeding				
Exclusive	133 (40.4)	163 (47.1)	296 (43.9)	<0.001
Mixed	129 (39.2)	173 (50.0)	302 (44.7)	
None	67 (20.4)	10 (2.9)	77 (11.4)	
Maternal recurrent respiratory symptoms/low lung function	25 (7.6)	31 (8.9)	56 (8.3)	0.522
Maternal HIV infected	120 (36.5)	9 (2.6)	129 (19.1)	<0.001
Maternal stress high	62 (18.8)	71 (20.5)	133 (19.7)	0.727
Maternal alcohol, high risk	8 (2.4)	21 (6.1)	29 (4.3)	0.066
Previous LRTI	12 (3.6)	23 (6.6)	35 (5.1)	0.077

LRTI, lower respiratory tract infection.

Despite an HIV prevalance of 19%, no infants were HIV infected due to a strong prevention of mother-to-child transmission programme that included antiretroviral therapy to all HIV-infected pregnant women and to infants for 6 weeks postpartum.

Successful measurements were obtained in 649 (95%) of tidal breathing, 614 (90%) MBW and 508/645 (79%) FOT measures (see online supplementary table S2).

### Associations of early lung function

The results of the univariate and multivariate analyses of associations with lung function measures are shown in online supplementary tables S3–S11, and the results of the multivariate analyses are summarised below.

#### Infant growth and lung maturation

Infant size, age and WAZ were associated with larger V_T_, with V_T_ increasing by 2.5 mL for every unit increase in WAZ (p<0.001; 95% CI 1.9 to 3.1). Birth weight z score was associated with lower respiratory rate (difference −3 bpm (95% CI −4 to −1), p<0.001), larger FRC (4 mL (1.8 to 6.1), p<0.001) and higher C_RS_ (0.06 mL/cmH_2_O (0.01 to 0.12), p=0.03). Gestational age was associated with a small but statistically significant decrease in respiratory rate (−1 bpm per week increase; (−1.4 to −0.1), p=0.02), increased V_T_ (0.4 mL (0.1 to 0.7), p=0.006), larger FRC (2.6 mL 90.3 to 2.2) p=0.01) and increased C_RS_ (0.03 mL/cmH_2_O (0.006 to 0.06), p=0.02) at 6–10 weeks of age. However, neither somatic growth at birth nor at testing nor gestational age was associated with the tidal expiratory flow ratio of time to peak tidal expiratory flow over total expiratory time (t_PTEF_/t_E_) and R_RS_.

#### Sex and ethnicity

Male infants had larger V_T_ compared with female infants (2.4 mL difference (95% CI 1.4 to 3.4), p<0.001) but lower t_PTEF_/t_E_ (−3.2% (−5.5 to −1.0), p=0.005), higher R_RS_ (4.1 cmH_2_O s/L (1.0 to 7.3), p=0.01) and lower C_RS_ (−0.12 mL/cmH_2_O (−0.2 to −0.03), p=0.008).

Black African infants had a higher respiratory rate (3.6 bpm difference (95% CI 1.05 to 6.1), p=0.006), increased t_PTEF_/t_E_ (4.4% (1.7 to 7.2), p=0.002) and increased inspiratory time over total breath time (t_I_/t_TOT_) (1.3% (0.2 to 2.4), p=0.02), but similar V_T_ compared with infants of mixed ethnicity. Ethnicity had no effect on other lung function outcomes measured (see online supplementary tables S2–S10).

#### Socioeconomic and environmental factors

Infants whose mothers smoked during pregnancy had lower V_T_ (−1.6 mL (95% CI −3.0 to −0.1), p=0.04) and higher LCI (0.1 FRC turnovers (0.01 to 0.3), p=0.03) compared with infants whose mothers did not smoke during pregnancy. The t_PTEF_/t_E_, FRC and C_RS_ were also lower in smoke-exposed compared with non-exposed infants, but these did not remain statistically significant after correcting for predictors and confounders. Infants living in homes with high levels of household benzene had lower t_PTEF_/t_E_ (−3.0% (−5.2 to −0.7), p=0.01) compared with infants from low-level households (see online supplementary figure S2). Household benzene exposure had no effect on other lung function outcomes. SES was not associated with lung function differences.

#### Maternal factors

High maternal distress scores, breastfeeding practises and history of maternal recurrent respiratory symptoms were not associated with lung function outcomes. Infants of HIV-infected mothers had higher V_T_ (1.7 mL (95% CI 0.06 to 3.3), p=0.04) compared with infants who were not HIV exposed, but no other differences in lung function were observed.

Infants whose mothers drank alcohol during pregnancy had lower respiratory rates (−7 bpm (−13 to −2), p=0.009), higher tidal volumes (3.0 mL (CI 0.4 to 5.5), p=0.02) and lower t_PTEF_/t_E_ (−10% (−15.4 to −3.7), p=0.002) compared with infants whose mothers did not drink alcohol (see online supplementary figure S3).

#### Previous LRTI

Thirty-five (5%) infants had an LRTI prior to lung function testing (table 2). These infants had similar lung function at 6 weeks to infants who had no LRTI after adjusting for confounding factors (see online supplementary tables S3–S11).

## Discussion

This is the first study from a low-middle income country setting to show several antenatal and early-life factors that impact on lung function, affecting both lung structure and control of breathing in 6-week-old African infants. Known factors such as infant growth, sex and maternal smoking and novel risk factors such as maternal alcohol ingestion and HIV exposure were identified.

We have published methodology using the FOT in this cohort, showing it to be sensitive enough to detect the impact of sex and antenatal smoke exposure on lung function^23^ and have defined normative data in these infants.^28^ This paper analyses the impact of antenatal factors on early lung growth and function in a large cohort of infants. Different antenatal and early-life exposures affected either lung structural development as evidenced by reduced lung volumes and lung compliance or control of breathing with effects on respiratory rate and flow ratios or both. Somatic growth, age and gestational age primarily affected lung development, while ethnicity, alcohol exposure, HIV exposure or benzene affected control of breathing, sex and tobacco smoke exposure affected both.

Infant size, in particularly length, is an important predictor of lung function outcomes early in life.^29–31^ As both weight and length at test were strongly associated with lung function outcomes in our study, we included only one measure of infant size, weight at test, in our model. As expected infant size at birth and a few weeks postnatally was associated with increased lung size and C_RS_ at 6 weeks. Size at birth is associated with early lung development.^32^ The fact that resistance was not associated with somatic growth in this study is likely related to the measurement method which, by using a mask over the mouth and nose, includes the resistance of the upper airway which is large and dominates the measured effect on the R_RS_.^33^

Our findings of sex differences in lung function are consistent with previous reports of low lung function in male infants,^34^ ^35^ which suggest that male infants have relatively smaller airways for lung size compared with female infants. Lower early lung function in boys may be one factor contributing to the higher rate of LRTI in boys.^36^

The effect of ethnic differences may be difficult to distinguish from sociocultural and environmental factors. However, even after adjusting for exposure differences, lung function remained different between black African and mixed ancestry infants. Black African infants had higher respiratory rate and higher flow ratios, but similar lung volumes and impedance to mixed ancestry infants, suggesting a different early pattern of breathing in African infants. This is consistent with previously described ethnic differences between black Afro-Caribbean and Caucasian European infants.^34^ ^37^ ^38^

Smoke-exposed infants had smaller lung volumes suggesting early structural lung impairment. Previous studies have shown that both in utero smoke exposure^37^ and early-life environmental exposure to tobacco smoke (ETS) impair early lung function.^8^ In this study, we were unable to distinguish between the effect on lung function of in utero or early-life smoke exposure as lung function was measured a few weeks after birth. As our cohort had an extremely high passive smoke exposure rate (75%), it is likely that some of the effects seen are due to ETS in early life. Although altered control of breathing has been described in smoke-exposed infants,^39^ our lung function measures may not be sensitive nor specific enough to detect these effects.

Infants exposed to high household benzene levels had lower t_PTEF_/t_E_ which may be related to airway obstruction or to breathing pattern. The t_I_/t_TOT_ more specifically reflects the central control of breathing, but was not associated with benzene exposure in this study. Further research evaluating the impact of antenatal and early exposure to indoor air pollutants on lung health is needed.

SES did not impact lung function outcomes. This may reflect the overall poor SES of the populations studied. Prior LRTI also did not affect lung function; however, the number of children with LRTI was very small, and the study was therefore not adequately powered to investigate this.

Consistent with several other studies, we found no association between maternal chronic respiratory illness and infant lung function.^40–42^

This is the first study to show an effect of alcohol exposure on infant lung function. The deleterious effect of alcohol exposure on the developing fetal brain is well understood and described, providing a possible basis for altered control of breathing in alcohol-exposed infants. In addition, in utero alcohol exposure reduces surfactant protein and alveolar macrophage function and increases lung susceptibility to trauma and infection in animal models.^43^ ^44^ Animal studies have also shown alcohol exposure during pregnancy to impact lung growth and structure,^45^ although some do not persist into early postnatal life.^46^ The number of alcohol-exposed infants included was small, and hence this result should be interpreted with caution and confirmed in a larger cohort. Further, in this study alcohol exposure may have been underestimated due to underreporting. However, this represents the minimum number of alcohol-exposed infants, with a very strong association for impaired lung function demonstrated. Further research of the potential mechanisms and impact is needed.

This is also the first study to show an association between HIV exposure and lung function, with exposed infants having higher V_T_, suggesting an effect on control of breathing. This effect may be mediated through HIV or due to antiretroviral therapy taken by mothers and infants with dysregulation of metabolic pathways. Further research is needed including long-term follow-up of infants, which we are currently undertaking.

Strengths of this study include the large sample size, the high success rate of testing, the comprehensive lung function testing that was done and the measurement of many, diverse potential risk factors that may impact on lung function. In addition, this is the first study to provide lung function measurements in African infants. A limitation is the possible lack of generalisability to other LMICs or to other ethnic groups. However, many of the risk factors described are highly prevalent in LMICs. In addition, our model had low R^2^ values (see online supplementary tables S1–S9), suggesting that there are other unaccounted for factors contributing to early-life lung function.

We identified several risk factors for impaired lung function that are amenable to public health interventions and several that require further study, including maternal HIV infection and use of alcohol during pregnancy. Avoidance of maternal smoking and smoking cessation programme for pregnant women should be a priority, and implementing strategies to reduce exposure to benzene should be undertaken in this population. Long-term study of lung function and respiratory health in these infants is a priority that we are undertaking to better understand the clinical implications of these findings and to inform new strategies to strengthen child health in LMICs.

## References

[1] Liu L, Oza S, Hogan D, et al Global, regional, and national causes of child mortality in 2000–13, with projections to inform post-2015 priorities: an updated systematic analysis. Lancet 2015;385:430–40. 10.1016/S0140-6736(14)61698-6 25280870

[2] Gold DR, Tager IB, Weiss ST, et al Acute lower respiratory illness in childhood as a predictor of lung function and chronic respiratory symptoms. Am Rev Respir Dis 1989;140:877–84. 10.1164/ajrccm/140.4.877 2802376

[3] Stern DA, Morgan WJ, Wright AL, et al Poor airway function in early infancy and lung function by age 22 years: a non-selective longitudinal cohort study. Lancet 2007;370:758–64. 10.1016/S0140-6736(07)61379-8 17765525 PMC2831283

[4] Hoo AF, Stocks J, Lum S, et al Development of lung function in early life: influence of birth weight in infants of nonsmokers. Am J Respir Crit Care Med 2004;170:527–33. 10.1164/rccm.200311-1552OC 15172896

[5] Jones M Effect of preterm birth on airway function and lung growth. Paediatr Respir Rev 2009;10(Suppl 1):9–11. 10.1016/S1526-0542(09)70005-3 19651391

[6] Clarke JR, Reese A, Silverman M Bronchial responsiveness and lung function in infants with lower respiratory tract illness over the first six months of life. Arch Dis Child 1992;67:1454–8. 10.1136/adc.67.12.1454 1489224 PMC1793966

[7] Dezateux C, Lum S, Hoo AF, et al Low birth weight for gestation and airway function in infancy: exploring the fetal origins hypothesis. Thorax 2004;59:60–6. 14694251 PMC1758850

[8] Stocks J, Dezateux C The effect of parental smoking on lung function and development during infancy. Respirology 2003;8:266–85. 10.1046/j.1440-1843.2003.00478.x 14528876

[9] Latzin P, Röösli M, Huss A, et al Air pollution during pregnancy and lung function in newborns: a birth cohort study. Eur Respir J 2009;33:594–603. 10.1183/09031936.00084008 19010988

[10] Zar HJ, Barnett W, Myer L, et al Investigating the early-life determinants of illness in Africa: the Drakenstein Child Health Study. Thorax 2015;70:592–4. 10.1136/thoraxjnl-2014-206242 25228292 PMC5107608

[11] Myer L, Stein DJ, Grimsrud A, et al Social determinants of psychological distress in a nationally-representative sample of South African adults. Soc Sci Med 2008;66:1828–40. 10.1016/j.socscimed.2008.01.025 18299167 PMC3203636

[12] WHO. Integrated management of childhood illness: distance learning course. Geneva: WHO, 2014.

[13] Quanjer PH, Stanojevic S, Cole TJ, et al Multi-ethnic reference values for spirometry for the 3–95-yr age range: the global lung function 2012 equations. Eur Respir J 2012;40:1324–43. 10.1183/09031936.00080312 22743675 PMC3786581

[14] Stein DJ, Koen N, Donald KA, et al Investigating the psychosocial determinants of child health in Africa: the Drakenstein Child Health Study. J Neurosci Methods 2015;252:27–35. 10.1016/j.jneumeth.2015.03.016 25797842 PMC4556362

[15] Harpham T, Reichenheim M, Oser R, et al Measuring mental health in a cost-effective manner. Health Policy Plan 2003;18:344–9. 10.1093/heapol/czg041 12917276

[16] Vanker A, Barnett W, Brittain K, et al Antenatal and early life tobacco smoke exposure in an African birth cohort study. Int J Tuberc Lung Dis 2016;20:729–37. 10.5588/ijtld.15.0697 27155174

[17] Vanker A, Barnett W, Nduru PM, et al Home environment and indoor air pollution exposure in an African birth cohort study. Sci Total Environ 2015;536:362–7. 10.1016/j.scitotenv.2015.06.136 26231768

[18] Frey U, Stocks J, Coates A, et al Specifications for equipment used for infant pulmonary function testing. ERS/ATS Task Force on Standards for Infant Respiratory Function Testing. European Respiratory Society/American Thoracic Society. Eur Respir J 2000;16:731–40. 11106221 10.1034/j.1399-3003.2000.16d28.x

[19] Robinson PD, Latzin P, Verbanck S, et al Consensus statement for inert gas washout measurement using multiple- and single-breath tests. Eur Respir J 2013;41:507–22. 10.1183/09031936.00069712 23397305

[20] Gray DM, Willemse L, Alberts A, et al Lung function in African infants: a pilot study. Pediatr Pulmonol 2015;50:49–54. 10.1002/ppul.22965 24339198 PMC4312776

[21] Hantos Z, Czövek D, Gyurkovits Z, et al Assessment of respiratory mechanics with forced oscillations in healthy newborns. Pediatr Pulmonol 2015;50:344–52. 10.1002/ppul.23103 25154334

[22] Schibler A, Hall GL, Businger F, et al Measurement of lung volume and ventilation distribution with an ultrasonic flow meter in healthy infants. Eur Respir J 2002;20:912–18. 10.1183/09031936.02.00226002 12412683

[23] Gray D, Czövek D, Smith E, et al Respiratory impedance in healthy unsedated South African infants: effects of maternal smoking. Respirology 2015;20:467–73. 10.1111/resp.12463 25581268 PMC4670479

[24] Textor J, Hardt J, Knüppel S DAGitty: a graphical tool for analyzing causal diagrams. Epidemiology 2011;22:745 10.1097/EDE.0b013e318225c2be 21811114

[25] Shrier I, Platt RW Reducing bias through directed acyclic graphs. BMC Med Res Methodol 2008;8:70 10.1186/1471-2288-8-70 18973665 PMC2601045

[26] The WHO Child Growth Standards. 2006 http://www.who.int/childgrowth/standards/en/

[27] Fenton TR, Kim JH A systematic review and meta-analysis to revise the Fenton growth chart for preterm infants. BMC Pediatr 2013;13:59 10.1186/1471-2431-13-59 23601190 PMC3637477

[28] Gray D, Willemse L, Visagie A, et al Lung function and exhaled nitric oxide in healthy unsedated African infants. Respirology 2015;20:1108–14. 10.1111/resp.12579 26134556 PMC4623783

[29] Hoo AF, Dezateux C, Hanrahan JP, et al Sex-specific prediction equations for Vmax(FRC) in infancy: a multicenter collaborative study. Am J Respir Crit Care Med 2002;165:1084–92. 10.1164/ajrccm.165.8.2103035 11956049

[30] Lum S, Stocks J, Stanojevic S, et al Age and height dependence of lung clearance index and functional residual capacity. Eur Respir J 2013;41:1371–7. 10.1183/09031936.00005512 23143552

[31] Nguyen TT, Hoo AF, Lum S, et al New reference equations to improve interpretation of infant lung function. Pediatr Pulmonol 2013;48:370–80. 10.1002/ppul.22656 22949414

[32] Lucas JS, Inskip HM, Godfrey KM, et al Small size at birth and greater postnatal weight gain: relationships to diminished infant lung function. Am J Respir Crit Care Med 2004;170:534–40. 10.1164/rccm.200311-1583OC 15172897

[33] Hall GL, Hantos Z, Wildhaber JH, et al Contribution of nasal pathways to low frequency respiratory impedance in infants. Thorax 2002;57:396–9. 10.1136/thorax.57.5.396 11978914 PMC1746337

[34] Stocks J, Henschen M, Hoo AF, et al Influence of ethnicity and gender on airway function in preterm infants. Am J Respir Crit Care Med 1997;156:1855–62. 10.1164/ajrccm.156.6.9607056 9412566

[35] Hanrahan JP, Brown RW, Carey VJ, et al Passive respiratory mechanics in healthy infants. Effects of growth, gender, and smoking. Am J Respir Crit Care Med 1996;154(Pt 1):670–80. 10.1164/ajrccm.154.3.8810604 8810604

[36] Zar HJ, Barnett W, Stadler A, et al Aetiology of childhood pneumonia in a well vaccinated South African birth cohort: a nested case-control study of the Drakenstein Child Health Study. Lancet Respir Med 2016;4:463–72. 10.1016/S2213-2600(16)00096-5 27117547 PMC4989125

[37] Hoo AF, Henschen M, Dezateux C, et al Respiratory function among preterm infants whose mothers smoked during pregnancy. Am J Respir Crit Care Med 1998;158:700–5. 10.1164/ajrccm.158.3.9711057 9730993

[38] Stocks J, Godfrey S Nasal resistance during infancy. Respir Physiol 1978;34:233–46. 10.1016/0034-5687(78)90031-2 705082

[39] Ueda Y, Stick SM, Hall G, et al Control of breathing in infants born to smoking mothers. J Pediatr 1999;135(Pt 1):226–32. 10.1016/S0022-3476(99)70026-0 10431118

[40] Young S, Sherrill DL, Arnott J, et al Parental factors affecting respiratory function during the first year of life. Pediatr Pulmonol 2000;29:331–40. 10.1002/(SICI)1099-0496(200005)29:5<331::AID-PPUL1>3.0.CO;2-A 10790244

[41] Tager IB, Hanrahan JP, Tosteson TD, et al Lung function, pre- and post-natal smoke exposure, and wheezing in the first year of life. Am Rev Respir Dis 1993;147:811–17. 10.1164/ajrccm/147.4.811 8466114

[42] Tepper RS, Reister T Forced expiratory flows and lung volumes in normal infants. Pediatr Pulmonol 1993;15:357–61. 10.1002/ppul.1950150608 8337014

[43] Gauthier TW, Young PA, Gabelaia L, et al In utero ethanol exposure impairs defenses against experimental group B streptococcus in the term Guinea pig lung. Alcohol Clin Exp Res 2009;33:300–6. 10.1111/j.1530-0277.2008.00833.x 19032578 PMC2662374

[44] Lazic T, Wyatt TA, Matic M, et al Maternal alcohol ingestion reduces surfactant protein A expression by preterm fetal lung epithelia. Alcohol 2007;41:347–55. 10.1016/j.alcohol.2007.07.006 17889311 PMC2083706

[45] Wang X, Gomutputra P, Wolgemuth DJ, et al Effects of acute alcohol intoxication in the second trimester of pregnancy on development of the murine fetal lung. Am J Obstet Gynecol 2007;197:269.e1–4. 10.1016/j.ajog.2007.06.031 17826415

[46] Sozo F, Vela M, Stokes V, et al Effects of prenatal ethanol exposure on the lungs of postnatal lambs. Am J Physiol Lung Cell Mol Physiol 2011;300:L139–47. 10.1152/ajplung.00195.2010 21036920 PMC3023283

