# Supplementary material for: Determinants of early-life lung function in African infants
Source: Thorax. 2016 Nov 17;72(5):445–50. doi: 10.1136/thoraxjnl-2015-207401 (PMC5520243; doi:10.1136/thoraxjnl-2015-207401)

On line supplement: Determinants of early life lung function in African infants

Diane Gray MD[1], Lauren Willemse B.Cur [1] , Ane Alberts B.Tech[1] , Dorottya Czövek MD[2], Polite Nduru Mphil[3], Aneesa Vanker MD[1], Dan J. Stein MD[4], Nastassja Koen MD[4], Peter D. Sly MD[2], Zoltán Hantos PhD[5], Graham L. Hall PhD[6#], Heather J. Zar MD[1#]

[1]Department of Paediatrics and Child Health, Red Cross War Memorial Children's Hospital and MRC Unit on Child and Adolescent Health, University of Cape Town, Cape Town, South Africa;

[2]Children's Lung, Environment and Asthma Research, Child Health Research Centre, Unviversity of Queensland, Brisbane, Australia;

[3]Division of Epidemiology and Biostatistics, Department of Public Health and Family Medicine, University of Cape Town, Cape Town, South Africa;

[4]Department of Psychiatry and MRC Unit on Anxiety and Stress Disorder, University of Cape Town, Cape Town, South Africa;

[5]Department of Medical Physics and Informatics, University of Szeged, Szeged, Hungary;

[6]Telethon Kids Institute, Perth, Australia Centre for Child Health Research, University of Western Australia, School of Physiotherapy and Exercise Science, Faculty of Health Sciences, Curtin University, Perth, Australia;

Table S1 Methodological detail of study measurements

Measurement	Definition	Collected
Birth-weight for gestational age z-score	Z-score calculated using the Fenton New-born growth charts	At birth
Weight and height for age z-scores	Z-scores calculated using the WHO Child Growth Standards. Age corrected for prematurity (<37 weeks)	On day of lung function test (6 week study visit)
Gestational age	Assessed by antenatal ultrasound or combination of last menstrual period, antenatal examination of pubic symphysis fundal height and birth weight, when ultrasound was not available	Antenatal 2nd trimester ultrasound/examination
Maternal smoking	Urine cotinine: the highest value from two visits used to classify as active smoker (cotinine >500 $ng.ml^{-1}$), passive smoker (10-500 $ng.ml^{-1}$) or non-smoker (<10 $ng.ml^{-1}$).[1]	Antenatal study visit (28-32 weeks gestation) and at birth; using the IMMULITE[R] 1000 Nicotine Metabolite Kit[1]
Household benzene	Categorized as above threshold if benzene >5mcg/m^3; based on the 2015 South African National Ambient Air Quality Standards (SANAAQ).	Antenatal home visit using a Markes[R] thermal desorption tube left in the home for two weeks
Socioeconomic status	Composite variable, placing participants into relative quartiles. Score is derived from employment status and standardized scores of: educational attainment, household income, assets and market access.[2]	Based on questionnaire information collected at antenatal study visit (28-32 weeks).
Maternal distress	Defined as high risk if WHO endorsed Self Reporting Questionaire (SRQ) score ≥8.	Antenatal study visit (28-32 wk), SRQ-20 questionnaire.[3, 4]
Maternal alcohol dependence/high risk	Daily and/or weekly use of alcohol in the preceding 3 months.	Antenatal visit (28-32 wk) Alcohol, Smoking and Substance Test questionnaire.[3, 4]
Maternal respiratory health	Maternal recurrent respiratory symptoms: chronic cough or recurrent wheeze in previous 12 months, doctor diagnosed asthma, bronchiectasis or chronic obstructive pulmonary disease ever. Low FEV_1: < -2 SD predicted using the Global Lung Initiative multi-ethnic equations[5]	Antenatal study visit (28-32 weeks) and 6 week visit questionnaire
Infant feeding	Exclusive breastfeeding: breast-milk only; Mixed feeding: mixture of breast-milk and infant formulas; No breastfeeding: infant formula only.	6 week study visit questionnaire, questions related to the previous 6-10 weeks feeding practices
HIV status	Mothers: point of care rapid HIV antibody test, positive test confirmed with laboratory based combined antibody and antigen test.<br>Infants of HIV positive mothers: HIV PCR test	Mothers: pregnancy booking and labour<br>Exposed infants: birth and 4 weeks

Table S2: Lung function outcomes

Lung function variable	Median	IQR	CV[#] mean (SD)
**Tidal breathing n=649**			
Respiratory rate n.min^{-1}	47	41; 55	8.8 (2.9)
Tidal volume mL	34·2	30·0; 38·6	8.8 (3.4)
t_{PTEF}/t_E[*] %	38·6	30·2; 46·2	23.0 (9.3)
t_I/t_{TOT}[*] %	45·2	42·0; 48·8	6.6 (2.4)
t_E/t_{TOT} [*]%	54·8	51·2; 58·0	5.5 (2.1)
**Multiple breath washout n=614**			
Functional residual capacity (FRC) mL	74·5	65·5; 86·3	5.8 (3.5)
Lung clearance index n turnovers	7·1	6·9; 7·4	4.3 (2.5)
**Forced oscillation technique n=508**			
Resistance (R_{rs}) cmH_2O.s.L^{-1}	45·0	37·5; 55·8	6.4 (4.2)
Compliance (C_{RS}) mL.cmH_2O^{-1}	0·88	0·67; 1·14	13.1 (12.4)

[#] Intra-subject coefficient of variation [*]t_{PTEF}/t_E: time to peak tidal expiratory flow over total expiratory time; t_I/t_{TOT}: inspiratory time over total breath time; t_E/t_{TOT}: expiratory time over total breath time

Table S3: Univariate and multivariate analysis of respiratory rate

	Univariate (n=654)				Multivariate model (n=459)					
	Coefficient	95% CI		p-value	Coefficient	95% CI		p-value	Adj R^2	Residual Std Dev
**Respiratory rate ($n.min^{-1}$)**									7%	10.94
**Infant growth and lung maturation**										
**Weight-for-age z score**	-1.621	-2.420	-0.822	<0.001	0.347	-0.962	1.656	0.603		
**Gestation**	-0.653	-1.091	-0.215	0.004	-0.949	-1.596	-0.302	0.004		
**Birth weight z score**	-1.937	-2.775	-1.099	<0.001	-2.717	-4.120	-1.314	<0.001		
**Gender and ethnicity**										
**Male gender**	0.381	-1.424	2.186	0.679	-1.109	-3.190	0.972	0.295		
**Ethnicity Black African**	2.764	0.974	4.555	0.003	3.580	1.047	6.113	0.006		
**Environmental and socioeconomic factors**										
**Smoking: Non smoker**										
**Passive**	-1.910	-4.288	0.468	0.115	-0.622	-3.294	2.050	0.647		
**Active**	-0.418	-2.936	2.099	0.744	1.869	-1.307	5.045	0.248		
**Benzene**	-1.025	-3.072	1.021	0.325	-1.779	-3.832	0.275	0.089		
**Social Economic Status: Highest quartile**										
**Lowest quartile**	0.375	-2.121	2.872	0.768						
**Low-moderate quartile**	1.380	-1.198	3.958	0.294						
**Moderate-high quartile**	0.058	-2.571	2.687	0.965						
**Maternal factors**										
**Maternal stress**	-1.050	-3.302	1.202	0.360						
**Feeding choice: Exclusive breastfeeding**										
**Mixed feeding**	-0.116	-2.035	1.803	0.906						
**No breastfeeding**	0.390	-2.581	3.361	0.797						
**Maternal recurrent respiratory symptoms, low FEV_1**	-0.674	-3.381	2.033	0.625						
**Maternal HIV infection**	0.699	-1.621	3.020	0.554	-0.214	-3.165	2.737	0.887		
**Maternal alcohol**	-2.692	-7.102	1.718	0.231	-7.199	-12.627	-1.771	0.009		
**Previous lower respiratory tract infection**	2.698	-1.301	6.697	0.186	2.797	-1.762	7.356	0.229		

Table S4: Univariate and multivariate analysis of tidal volume

	Univariate (n=654)				Multivariate model (n=459)					
	Coefficient	95% CI		p-value	Coefficient	95% CI		p-value	Adj R^2	Residual Std Dev
**Tidal volume (mL)**									33%	5.14
**Infant growth and lung maturation**										
**Weight-for-age z score**	2.863	2.487	3.239	**<0.001**	2.492	1.876	3.109	**<0.001**		
**Gestation**	0.670	0.438	0.902	**<0.001**	0.454	0.149	0.759	**0.004**		
**Birth weight z score**	1.803	1.368	2.239	**<0.001**	0.492	-0.171	1.155	0.146		
**Gender and ethnicity**										
**Male gender**	1.323	0.356	2.289	**0.007**	2.416	1.438	3.395	**<0.001**		
**Ethnicity**	1.076	0.109	2.043	**0.029**	-0.804	-2.011	0.403	0.191		
**Environmental and socioeconomic factors**										
**Smoking: Non smoker**										
**Passive**	-0.170	-1.446	1.107	0.794	-0.553	-1.811	0.705	0.388		
**Active**	-2.491	-3.842	-1.139	**<0.001**	-1.549	-3.045	-0.053	**0.042**		
**Benzene**	0.467	-0.648	1.583	0.411	0.803	-0.168	1.775	0.105		
**Social Economic Status: highest quartile**										
**Lowest quartile**	-1.059	-2.401	0.283	0.122						
**Low-moderate quartile**	-0.070	-1.456	1.316	0.921						
**Moderate-high quartile**	0.171	-1.242	1.584	0.812						
**Maternal factors**										
**Maternal stress**	-0.599	-1.800	0.603	0.328						
**Feeding choice: Exclusive breastfeeding**										
**Mixed feeding**	0.577	-0.452	1.606	0.271	0.747	-0.279	1.773	0.153		
**No breastfeeding**	1.746	0.153	3.339	**0.032**	1.499	-0.351	3.349	0.112		
**Maternal recurrent respiratory symptoms, low FEV_1**	0.256	-1.201	1.713	0.731						
**Maternal HIV infection**	1.686	0.442	2.929	0.008	1.679	0.059	3.300	**0.042**		
**Maternal alcohol**	-1.118	-3.472	1.237	0.352	2.955	0.403	5.506	**0.023**		
**Previous lower respiratory tract infection**	-2.033	-4.182	0.117	0.064	-1.025	-3.170	1.120	0.348		

Table S5: Univariate and multivariate analysis of the ratio of time to peak tidal expiratory flow over total expiratory time (tPTEF/tE)

	Univariate (n=654)				Multivariate model (n=459)					
	**Coefficient**	**95% CI**		**p-value**	**Coefficient**	**95% CI**		**p-value**	**Adj R^2**	**Residual Std Dev**
**t_{PTEF}/t_E (%)**									10%	11.87
**Infant growth and lung maturation**										
**Weight-for-age z score**	0.023	-0.833	0.880	0.957	-0.913	-2.327	0.500	0.205		
**Gestation**	0.379	-0.087	0.845	0.111	0.287	-0.412	0.987	0.420		
**Birth weight z score**	-0.021	-0.922	0.880	0.964	0.602	-0.920	2.123	0.437		
**Gender and ethnicity**										
**Male gender**	-2.750	-4.650	-0.850	**0.005**	-3.196	-5.440	-0.952	**0.005**		
**Ethnicity**	3.971	2.087	5.854	**<0.001**	4.414	1.648	7.179	**0.002**		
**Environmental and socioeconomic factors**										
**Smoking: Non smoker**										
**Passive**	-2.014	-4.543	0.514	0.118	-0.127	-3.012	2.758	0.931		
**Active**	-3.139	-5.815	-0.462	**0.022**	-0.036	-3.465	3.393	0.984		
**Benzene**	-1.706	-3.893	0.482	0.126	-2.931	-5.158	-0.704	**0.010**		
**Social Economic Status: highest quartile**										
**Lowest quartile**	0.764	-1.881	3.410	0.571						
**Low-moderate quartile**	-0.364	-3.095	2.367	0.794						
**Moderate-high quartile**	0.399	-2.387	3.184	0.779						
**Maternal factors**										
**Maternal stress**	0.774	-1.629	3.177	0.527						
**Feeding choice: Exclusive breastfeeding**										
**Mixed feeding**	-2.322	-4.333	-0.311	**0.024**	-2.192	-4.544	0.160	0.068		
**No breastfeeding**	3.018	-0.096	6.132	0.057	1.086	-3.158	5.330	0.615		
**Maternal recurrent respiratory symptoms, low FEV_1**	-2.419	-5.278	0.441	0.097	-2.422	-5.816	0.973	0.162		
**Maternal HIV infection**	3.218	0.773	5.664	**0.010**	-1.503	-5.232	2.225	0.429		
**Maternal alcohol**	-6.201	-10.879	-1.523	**0.009**	-8.485	-14.359	-2.612	**0.005**		
**Previous lower respiratory tract infection**	-3.984	-8.211	0.243	0.065	-1.647	-6.569	3.275	0.511		

Table S6: Univariate and multivariate analysis of the ratio of inspiratory time over total breath time (t_I/t_{TOT})

	Univariate (n=654)				Multivariate model (n=457)					
	Coefficient	95% CI		p-value	Coefficient	95% CI		p-value	Adj R^2	Residual Std Dev
**t_I/t_{TOT} (%)**									3%	4.60
**Infant growth and lung maturation**										
**Weight-for-age z score**	-0.245	-0.575	0.085	0.145	-0.016	-0.564	0.531	0.954		
**Gestation**	-0.150	-0.329	0.029	0.100	-0.086	-0.357	0.184	0.532		
**Birth weight z score**	-0.293	-0.636	0.051	0.095	-0.351	-0.938	0.237	0.241		
**Gender and ethnicity**										
**Male gender**	-0.436	-1.167	0.295	0.242	-0.542	-1.412	0.329	0.222		
**Ethnicity**	1.697	0.977	2.416	**<0.001**	1.275	0.203	2.348	**0.020**		
**Environmental and socioeconomic factors**										
**Smoking: Non smoker**										
**Passive**	-0.437	-1.392	0.517	0.369	-0.106	-1.224	1.012	0.852		
**Active**	-0.852	-1.863	0.159	0.098	-0.260	-1.589	1.069	0.701		
**Benzene**	0.049	-0.778	0.875	0.908	-0.213	-1.072	0.647	0.627		
**Social Economic Status: highest quartile**										
**Lowest quartile**	0.356	-0.651	1.364	0.488						
**Low-moderate quartile**	0.045	-1.001	1.091	0.933						
**Moderate-high quartile**	0.587	-0.478	1.653	0.279						
**Maternal factors**										
**Maternal stress**	-0.634	-1.561	0.294	0.180						
**Feeding choice: Exclusive**										
**Mixed feeding**	-0.089	-0.866	0.687	0.822						
**None**	0.090	-1.126	1.306	0.885						
**Maternal recurrent respiratory symptoms, low FEV_1**	-0.540	-1.640	0.560	0.336						
**Maternal HIV infection**	0.434	-0.507	1.374	0.366	-0.389	-1.623	0.846	0.537		
**Maternal alcohol**	-0.565	-2.348	1.218	0.534	-1.499	-3.770	0.773	0.195		
**Previous lower respiratory tract infection**	0.167	-1.462	1.796	0.841	0.964	-0.943	2.872	0.321		

Table S7: Univariate (n=654) and multivariate (n=459) analysis of the ratio of expiratory time over total breath time (t_E/t_{TOT})

	Univariate (n=654)				Multivariate (n=459) model					
	Coefficient	95% CI		p-value	Coefficient	95% CI		p-value	Adj R^2	Residual Std Dev
**t_E/t_{TOT} (%)**									3%	4.620
**<u>Infant growth and lung maturation</u>**										
**Weight-for-age z score**	0.245	-0.085	0.575	0.145	0.016	-0.531	0.564	0.954		
**Gestation**	0.150	-0.029	0.329	0.100	0.086	-0.184	0.357	0.532		
**Birth weight z score**	0.293	-0.051	0.636	0.095	0.351	-0.237	0.938	0.241		
**<u>Gender and ethnicity</u>**										
**Male gender**	0.436	-0.295	1.167	0.242	0.542	-0.329	1.412	0.222		
**Ethnicity**	-1.697	-2.416	-0.977	**<0.001**	-1.275	-2.348	-0.203	**0.020**		
**<u>Environment and socioeconomic factors</u>**										
**Smoking: Non smoker**										
**Passive**	0.437	-0.517	1.392	0.369	0.106	-1.012	1.224	0.852		
**Active**	0.852	-0.159	1.863	0.098	0.260	-1.069	1.589	0.701		
**Benzene**	-0.049	-0.875	0.778	0.908	0.213	-0.647	1.072	0.627		
**Social Economic Status: highest quartile 4**										
**Lowest quartile**	-0.356	-1.364	0.651	0.488						
**Low-moderate quartile**	-0.045	-1.091	1.001	0.933						
**Moderate-high quartile**	-0.587	-1.653	0.478	0.279						
**<u>Maternal factors</u>**										
**Maternal stress**	0.634	-0.294	1.561	0.180						
**Feeding choice: Exclusive**										
**Mixed feeding**	0.089	-0.687	0.866	0.822						
**None**	-0.090	-1.306	1.126	0.885						
**Maternal recurrent respiratory symptoms, low FEV_1**	0.540	-0.560	1.640	0.336						
**Maternal HIV infection**	-0.434	-1.374	0.507	0.366	0.389	-0.846	1.623	0.537		
**Maternal alcohol**	0.565	-1.218	2.348	0.534	1.499	-0.773	3.770	0.195		
**<u>Previous lower respiratory tract infection</u>**	-0.167	-1.796	1.462	0.841	-0.964	-2.872	0.943	0.321		

Table S8: Univariate and multivariate (n=435) analysis of the functional residual capacity (FRC)

	Univariate (n=563)				Multivariate model (n=435)					
	Coefficient	95% CI		p-value	Coefficient	95% CI		p-value	Adj R^2	Residual Std Dev
**FRC (mL)**									10%	15.90
**Infant growth and lung maturation**										
**Weight-for-age z score**	3.645	2.526	4.765	**<0.001**	0.228	-1.731	2.187	0.819		
**Gestation**	1.205	0.593	1.817	**<0.001**	1.308	0.350	2.266	**0.008**		
**Birth weight z score**	2.981	1.802	4.160	**<0.001**	3.965	1.839	6.091	**<0.001**		
**Gender and ethnicity**										
**Male gender**	-2.338	-4.880	0.205	0.071	-0.194	-3.323	2.936	0.903		
**Ethnicity**	2.710	0.173	5.248	**0.036**	2.764	-1.214	6.742	0.173		
**Environmental and socioeconomic factors**										
**Smoking: Non smoker**										
**Passive**	-1.987	-5.355	1.381	0.247	-1.995	-6.037	2.046	0.332		
**Active**	-4.676	-8.228	-1.124	**0.010**	-2.684	-7.521	2.154	0.276		
**Benzene**	-0.517	-3.516	2.482	0.735	-0.436	-3.517	2.645	0.781		
**Social Economic Status: highest quartile**										
**Lowest quartile**	-3.658	-7.177	-0.138	**0.042**	-2.540	-7.138	2.057	0.278		
**Low-moderate quartile**	-2.770	-6.401	0.861	0.135	-0.696	-5.169	3.777	0.760		
**Moderate-high quartile**	-3.726	-7.420	-0.031	**0.048**	-2.925	-7.403	1.553	0.200		
**Maternal factors**										
**Maternal stress**	-1.365	-4.571	1.841	0.403						
**Feeding choice: Exclusive**										
**Mixed feeding**	-0.027	-2.736	2.681	0.984						
**None**	2.736	-1.435	6.907	0.198						
**Maternal recurrent respiratory symptoms, low FEV_1**	0.670	-3.144	4.484	0.730						
**Maternal HIV infection**	2.415	-0.833	5.663	0.145	0.556	-3.850	4.962	0.804		
**Maternal alcohol**	1.012	-5.345	7.369	0.755	5.298	-2.869	13.466	0.203		
**Previous lower respiratory tract infection**	-3.207	-8.867	2.453	0.266	0.269	-6.674	7.212	0.939		

Table S9: Univariate and multivariate analysis of the lung clearance index (LCI)

	Univariate (n=563)				Multivariate model (n=435)					
	**Coefficient**	**95% CI**		**p-value**	**Coefficient**	**95% CI**		**p-value**	**Adj R^2**	**Residual Std Dev**
**LCI (n FRC turnovers)**									4%	0.44
**Infant growth and lung function**										
**Weight-for-age z score**	-0.012	-0.044	0.020	0.456	-0.015	-0.069	0.039	0.585		
**Gestation**	-0.007	-0.024	0.010	0.404	0.006	-0.021	0.032	0.667		
**Birth weight z score**	0.006	-0.027	0.039	0.739	0.013	-0.045	0.071	0.664		
**Gender and ethnicity**										
**Male gender**	0.035	-0.035	0.105	0.324	0.069	-0.016	0.154	0.111		
**Ethnicity**	0.007	-0.062	0.077	0.837	0.085	-0.020	0.189	0.114		
**Environmental and socioeconomic factors**										
**Smoking: Non smoker**										
**Passive**	0.060	-0.031	0.151	0.196	0.057	-0.052	0.166	0.304		
**Active**	0.113	0.017	0.209	**0.021**	0.139	0.011	0.268	**0.034**		
**Benzene**	0.071	-0.011	0.152	0.088	0.054	-0.031	0.138	0.213		
**Social Economic Status: Highest quartile**										
**Lowest quartile**	0.000	-0.097	0.096	0.996						
**Low-moderate quartile**	0.035	-0.065	0.135	0.491						
**Moderate-high quartile**	0.029	-0.073	0.130	0.579						
**Maternal factors**										
**Maternal stress**	0.057	-0.030	0.145	0.198						
**Feeding choice: Exclusive breastfeeding**										
**Mixed feeding**	-0.023	-0.097	0.051	0.540						
**No breastfeeding**	-0.012	-0.126	0.103	0.840						
**Maternal recurrent respiratory symptoms, low FEV_1**	0.011	-0.093	0.116	0.832						
**Maternal HIV infection**	-0.005	-0.094	0.084	0.908	-0.031	-0.151	0.090	0.614		
**Maternal alcohol**	-0.093	-0.266	0.080	0.290	-0.222	-0.446	0.002	0.052		
**Previous lower respiratory tract infection**	0.105	-0.050	0.260	0.182	0.120	-0.070	0.310	0.214		

Table S10: Univariate and multivariate analysis of resistance (R_{RS})

Univariate (n=568)					Multivariate model (n=393)					
	**Coefficient**	**95% CI**		**p-value**	**Coefficient**	**95% CI**		**p-value**	**Adj R^2**	**Residual Std Dev**
**R_{RS} $cmH_2O.L.s^{-1}$**									4%	15.33
**<u>Infant growth and lung maturation</u>**										
**Weight-for-age z score**	0.077	-1.059	1.212	0.895	0.858	-1.115	2.832	0.393		
**Gestation**	-0.391	-1.021	0.239	0.224	-0.778	-1.787	0.231	0.130		
**Birth weight z score**	-0.778	-1.964	0.407	0.198	-1.537	-3.644	0.571	0.152		
**<u>Gender and ethnicity</u>**										
**Male gender**	3.491	0.963	6.020	**0.007**	4.137	1.005	7.270	**0.010**		
**Ethnicity**	-0.820	-3.368	1.728	0.528	-0.952	-4.821	2.918	0.629		
**<u>Environmental and socioeconomic factors</u>**										
**Smoking: Non smoker**										
**Passive**	0.583	-2.847	4.012	0.739	-1.549	-5.626	2.528	0.456		
**Active**	-0.372	-3.971	3.227	0.839	-4.716	-9.533	0.100	0.055		
**Benzene**	-0.019	-3.013	2.976	0.990	0.412	-2.676	3.500	0.793		
**Social Economic Status: highest quartile**										
**Lowest quartile**	0.175	-3.342	3.692	0.922						
**Low-moderate quartile**	-1.444	-5.063	2.175	0.434						
**Moderate-high quartile**	-0.033	-3.757	3.690	0.986						
**<u>Maternal factors</u>**										
**Maternal stress**	-0.592	-3.748	2.564	0.713						
**Feeding choice: Exclusive breastfeeding**										
**Mixed feeding**	-2.151	-4.833	0.532	0.116						
**No breastfeeding**	-2.731	-7.111	1.649	0.221						
**Maternal recurrent respiratory symptoms, low FEV_1**	3.117	-0.652	6.886	0.105						
**Maternal HIV infection**	-1.853	-5.125	1.420	0.267	-1.356	-5.833	3.121	0.552		
**Maternal alcohol**	0.300	-5.825	6.424	0.923	2.098	-5.710	9.905	0.598		
**<u>Previous lower respiratory tract infection</u>**	1.211	-4.873	7.295	0.696	-3.459	-10.992	4.075	0.367		

Table S11: Univariate and multivariate analysis of tidal compliance (C_{RS})

	Univariate (n=568)				Multivariate model (n=393)					
	Coefficient	95% CI		p-value	Coefficient	95% CI		p-value	Adj R²	Residual Std Dev
**C_{RS} (mL.cmH$_2$0^{-1})**									4%	0.42
**Infant growth and lung maturation**										
**Weight-for-age z score**	0.037	0.008	0.066	**0.014**	-0.0254	-0.0791	0.0282	0.352		
**Gestation**	0.029	0.013	0.046	**<0.001**	0.0332	0.0058	0.0605	**0.017**		
**Birth weight z score**	0.033	0.002	0.064	**0.037**	0.0620	0.0047	0.1194	**0.034**		
**Gender and ethnicity**										
**Male gender**	-0.101	-0.166	-0.036	**0.003**	-0.1162	-0.2018	-0.0305	**0.008**		
**Ethnicity**	0.046	-0.020	0.112	0.169	-0.0258	-0.1361	0.0844	0.645		
**Environmental and socioeconomic factor**										
**Smoking: Non smoker**										
**Passive**	-0.030	-0.119	0.059	0.504	0.0108	-0.1024	0.1240	0.852		
**Active**	-0.117	-0.210	-0.024	**0.014**	-0.0692	-0.2037	0.0653	0.312		
**Benzene**	0.076	-0.004	0.156	0.063	0.0679	-0.0163	0.1522	0.114		
**Social Economic Status: Highest quartile**										
**Lowest quartile**	-0.092	-0.182	-0.001	**0.048**	-0.0727	-0.2002	0.0547	0.263		
**Low-moderate quartile**	-0.049	-0.143	0.044	0.298	-0.0344	-0.1545	0.0857	0.574		
**Moderate-high quartile**	-0.005	-0.101	0.091	0.918	0.0310	-0.0924	0.1543	0.622		
**Maternal factors**										
**Maternal stress**	-0.026	-0.108	0.056	0.534						
**Feeding choice: Exclusive breastfeeding**										
**Mixed feeding**	-0.002	-0.072	0.067	0.948						
**No breastfeeding**	0.074	-0.040	0.187	0.204						
**Maternal recurrent respiratory symptoms, low FEV$_1$**	-0.022	-0.120	0.075	0.651						
**Maternal HIV infection**	0.054	-0.031	0.138	0.213	0.0570	-0.0650	0.1790	0.359		
**Maternal alcohol**	-0.096	-0.255	0.063	0.235	-0.0834	-0.2955	0.1286	0.440		
**Previous lower respiratory tract infection**	-0.212	-0.369	-0.056	**0.008**	-0.0951	-0.3001	0.1099	0.362		

References:

1 Siemens. Immulite 1000 Nicotine Metabolite. UK, 2009.

2 Myer L, Stein DJ, Grimsrud A, Seedat S, Williams DR. Social determinants of psychological distress in a nationally-representative sample of South African adults. Soc Sci Med. 2008; **66**: 1828-40.

3 Stein DJ, Koen N, Donald KA, Adnams CM, Koopowitz S, Lund C, Marais A, Myers B, Roos A, Sorsdahl K, Stern M, Tomlinson M, van der Westhuizen C, Vythilingum B, Myer L, Barnett W, Brittain K, Zar HJ. Investigating the psychosocial determinants of child health in Africa: The Drakenstein Child Health Study. Journal of neuroscience methods. 2015.

4 Harpham T, Reichenheim M, Oser R, Thomas E, Hamid N, Jaswal S, Ludermir A, Aidoo M. Measuring mental health in a cost-effective manner. Health policy and planning. 2003; **18**: 344-9.

5 Quanjer PH, Stanojevic S, Cole TJ, Baur X, Hall GL, Culver BH, Enright PL, Hankinson JL, Ip MS, Zheng J, Stocks J, Initiative ERSGLF. Multi-ethnic reference values for spirometry for the 3-95-yr age range: the global lung function 2012 equations. Eur Respir J. 2012; **40**: 1324-43.

FIGURE S1: Directed acyclic graph constructed to select minimum set of adjustment variables

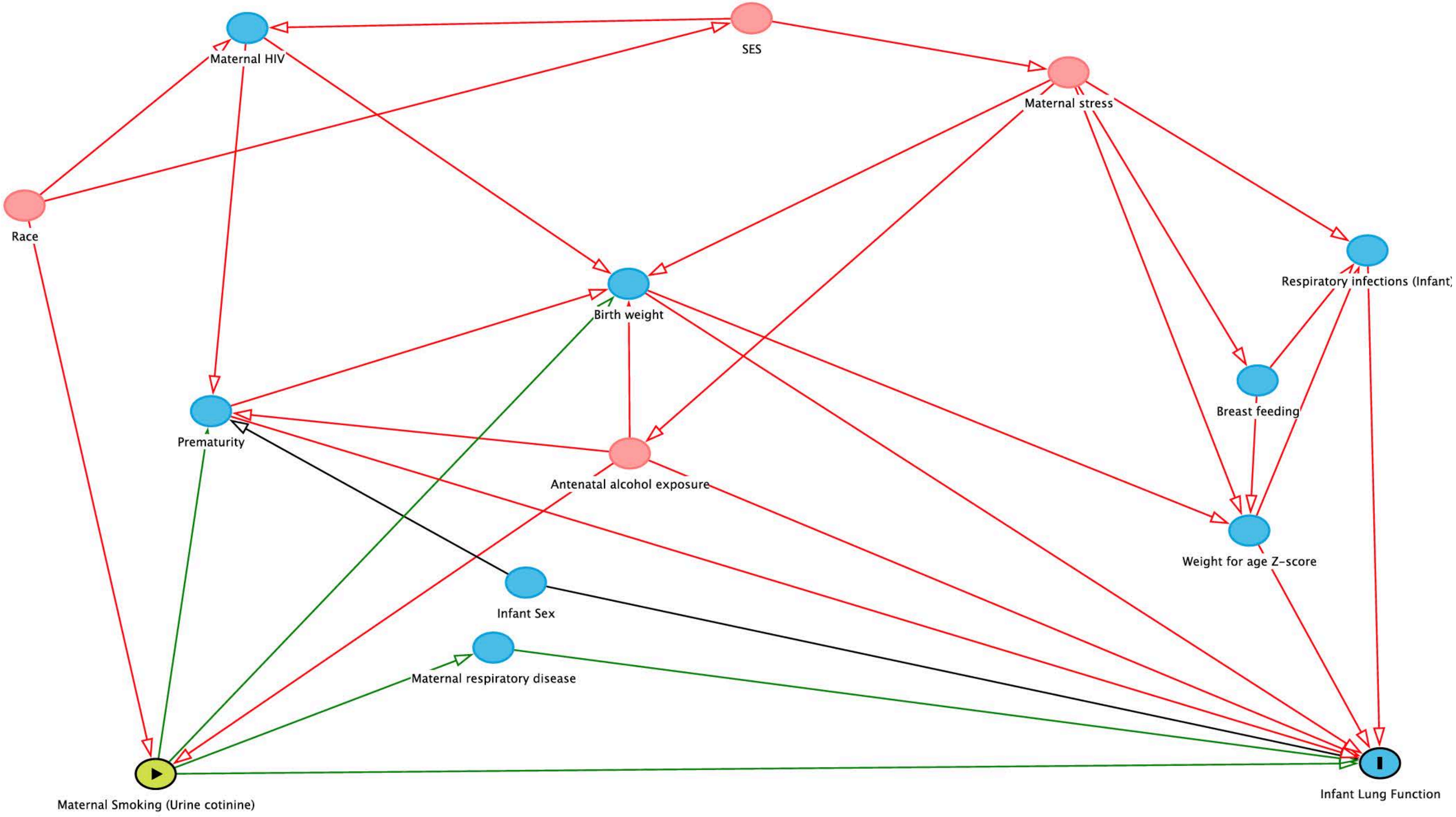

FIGURE S2: Household benzene exposure and ratio of time to peak tidal expiratory flow over total expiratory time, t_{PTEF}/t_E,

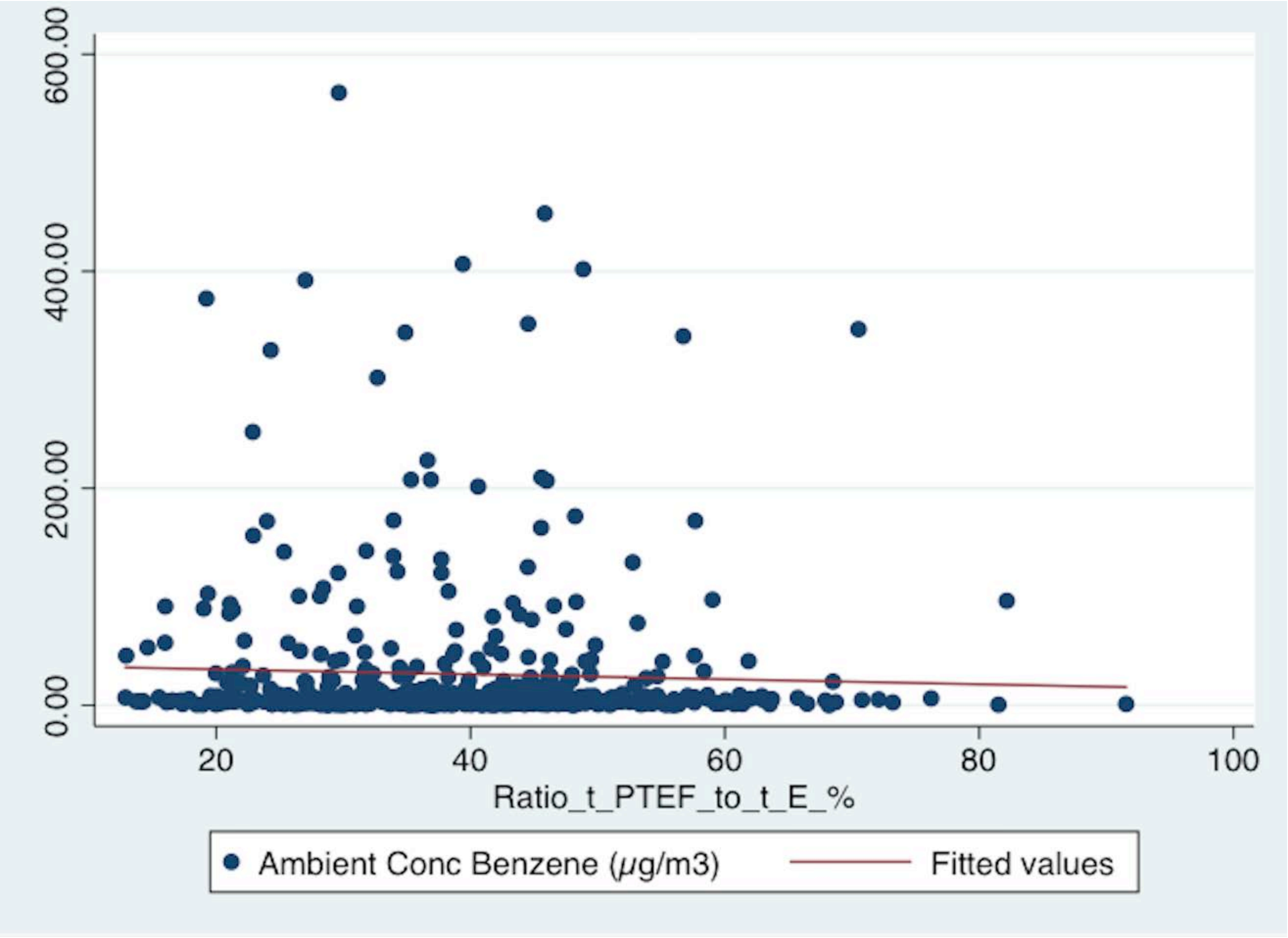

FIGURE S3: Alcohol exposure and tidal volume (top left), respiratory rate (top right) and ratio of time to peak tidal expiratory flow over total expiratory time, t_{PTEF}/t_E, (bottom)

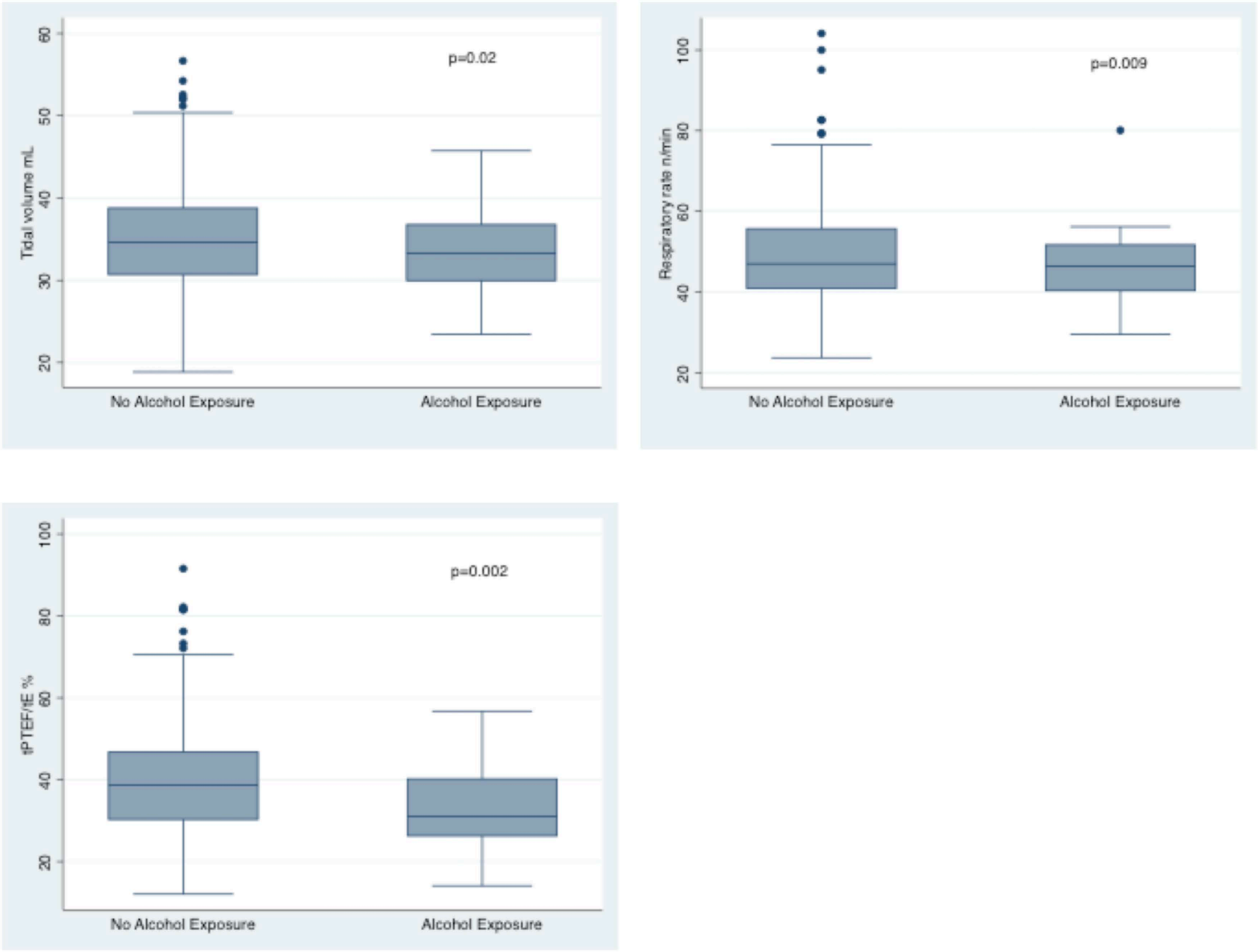

Supplement: supplementary data [file thoraxjnl-2015-207401supp001.pdf]
